# A Standardized Ward Round Proforma Improves Documentation in a Specialist Stroke Unit

**DOI:** 10.7759/cureus.31931

**Published:** 2022-11-27

**Authors:** Edward J Armstrong, Kilda J Carpenter

**Affiliations:** 1 Medical Education, The University of Buckingham, Buckingham, GBR; 2 Old Age Psychiatry, Oxford Health NHS Foundation Trust, Oxford, GBR

**Keywords:** checklist approach, quality improvement projects, mdt, medical documentation, ward round

## Abstract

Background and aim

Ward-round documentation is important for clinical communication and patient safety. Standardized checklists have improved ward-round documentation in surgical and medical settings. This quality improvement project aimed to introduce a standardized ward round proforma to improve documentation in a UK specialist stroke unit.

Methods

Ward round entries were assessed against internally agreed standardized criteria. A stroke-specific ward round proforma was designed and introduced with input from the multidisciplinary team. A repeat audit was performed, including assessment of the use of different proforma sections. Multidisciplinary team members were invited to provide feedback via an anonymous online survey.

Results

A total of 111 ward round entries were reviewed before the proforma was introduced. Ninety-five ward round entries were reviewed following introduction of the proforma, and 84.2% of these used the proforma for documentation. Overall documentation of standardized criteria improved from 48.7% to 62.1% with substantial improvement seen in documentation of neurological examination, presence/absence of mechanical venous thromboembolism prophylaxis, and blood test results. Multidisciplinary team feedback was positive.

Conclusions

The stroke-specific ward round proforma improved the quality and consistency of documentation in the unit. An updated proforma was designed using these results and multidisciplinary team feedback.

## Introduction

Ward rounds are a focal point of hospital care facilitating multidisciplinary review of patients and management planning. They serve as a vehicle for communication within the multidisciplinary team (MDT) and between the clinical team and patients. The Royal College of Physicians (RCP) considers clear documentation of ward rounds "essential" for communication [1]. Ward round notes facilitate continuity of care and are often the only written correspondence between responsible medical teams [2]. They also provide opportunity to summarize clinical information drawn from different papers and electronic sources [1].

Structured ward round approaches, including standardized checklists, are recommended by the National Institute for Health and Care Excellence (NICE), along with other professional bodies, as an intervention to improve patient outcomes [1,3]. Extensive published evidence supports the utility of written ward-round proformas in improving the quality and consistency of documentation in surgical units, with fewer studies demonstrating direct improvement in patient outcomes, including a reduction in prescription errors [4].

Similarly, multiple studies have demonstrated improvement in documentation, when measured against set criteria, following the introduction of a proforma for medical post-take ward rounds [5-7]. Other reported benefits of ward-round checklists in medical settings include a reduction in the time spent in documentation by junior medical staff [8] and an increase in the initiation of comprehensive geriatric assessment following admission [9].

Stroke is the leading cause of death and adult disability worldwide and occurs over 150,000 times a year in the United Kingdom (UK) [10]. The National Health Service (NHS) recommends people with strokes are managed in specialist stroke units, incorporating hyperacute stroke services facilitating access to prompt expert assessment and management [11]. Specialist care in a stroke unit has been associated with better outcomes and reduced death and dependency following stroke [12].

There is limited evidence to support the use of structured checklists in specialist stroke units. A 2015 study introduced an admission proforma in a stroke unit and found an improvement in the quality of clerking when measured against the RCP acute stroke management guidelines [13]. Another study introduced a standardized form for documenting MDT meetings and demonstrated an increase in the documentation of "needs" including bowel, urinary, and mood issues [14]. The proforma also improved goal setting and MDT communication. These studies, however, did not introduce standardized checklists for use on regular medical ward rounds.

Wycombe General Hospital (WGH) houses a regional specialist stroke unit incorporating a hyper acute stroke unit (HASU) and a specialist rehabilitation unit. This quality improvement (QI) project aimed to introduce a ward round proforma to improve the consistency and quality of documentation in WGH Stroke Unit.

## Materials and methods

Audit standard

The audit was approved by the Stroke Unit in Buckinghamshire Healthcare NHS Trust. The audit standard was a list of documentation parameters devised through internal consensus (Table 1). These were generated using the RCP’s "modern ward rounds" individual patient review recommendations and MDT discussion with input from consultants, registrars, junior medical staff, nursing staff, occupational therapists, and physiotherapists [1]. Patients and caregivers were not involved in the consultation process.

**Table 1:** Consensus criteria for stroke ward round documentation.

Consensus criteria for stroke ward round documentation
Time
Date
Day of admission
Clinician leading the ward round
Patient’s current issues/diagnosis
Observations
Blood test results
Investigation results
Evidence of drug chart review (including pharmacological venous thromboembolism {VTE} prophylaxis)
General examination
Neurological examination (for first seven days following admission)
Presence/absence of mechanical VTE prophylaxis
Hydration status
Nutrition status
Bowel function
Catheter plan
Discharge plan

Proforma design

**Figure 1:**
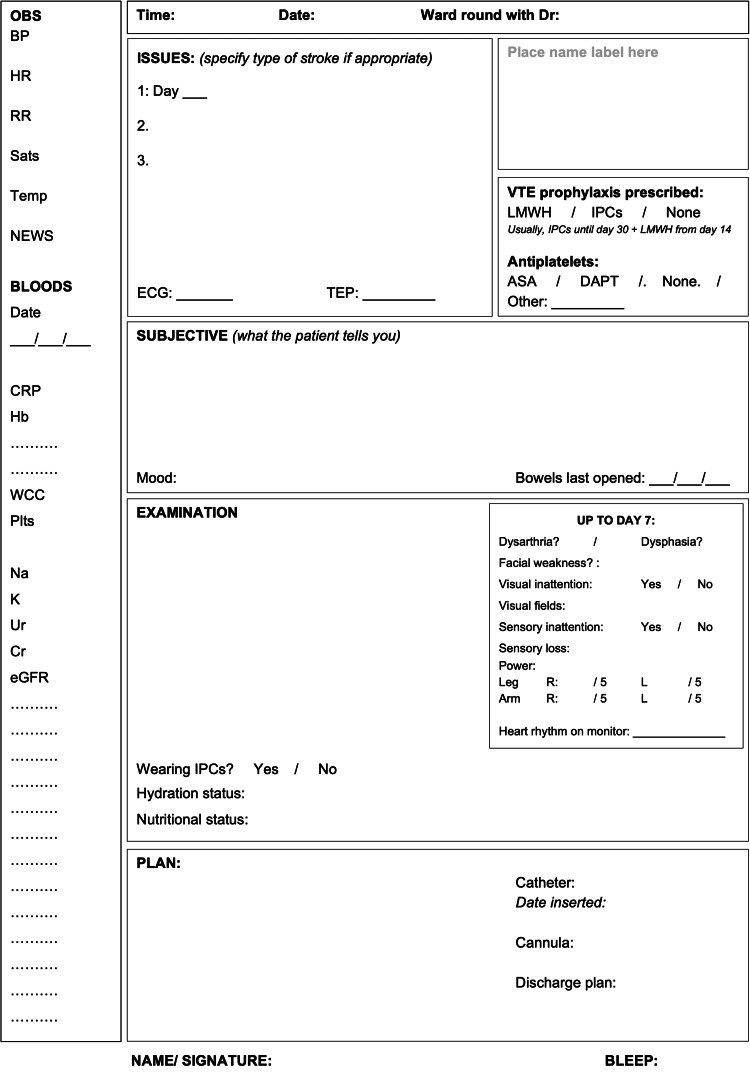
Ward round proforma design. OBS: observations; BP: blood pressure; HR: heart rate; RR: respiratory rate; Sats: oxygen saturations; Temp: temperature; NEWS: national early warning score; CRP: c-reactive protein; Hb: hemoglobin; WCC: white cell count; Plts: platelets; Na: sodium; K: potassium; Ur: urea; Cr: creatinine; eGFR: estimated glomerular filtration rate; LMWH: low molecular weight heparin; VTE: venous thromboembolism; IPCs: intermittent pneumatic compression device; ASA: aspirin; DAPT: dual antiplatelet therapy; ECG: electrocardiogram; TEP: treatment escalation plan; R: right; L: left

An initial draft of the ward round proforma was designed considering the consensus documentation criteria. The layout was modeled on a previously existing medical ward round proforma used in the same NHS Trust. Feedback was obtained through discussion with MDT stakeholders (as previously described) and the proforma was re-drafted multiple times in an iterative process. Wide-ranging changes were made, including adding dedicated space to document mood, electrocardiogram (ECG) results, cannula plan and current treatment escalation plan (TEP), items not included in the initial documentation consensus criteria (Figure 1). The formal TEP was documented elsewhere in the patient notes on a pre-existing trust-wide proforma; the TEP section on the proforma was intended to prompt clinicians to complete and review the TEP.

The proforma design was circulated electronically to junior medical staff and administrative staff to ensure printed copies were available on the ward. Documentation on daily medical ward rounds was performed primarily by junior medical staff who were encouraged, but not mandated, to use the proforma.

Sampling of clinical notes

All patients admitted to the stroke unit between February 1, 2021, and February 15, 2021, were identified and their retrospectively uploaded paper clinical notes were accessed electronically. A maximum of three consecutive ward round entries were reviewed for each patient. Documentation of the consensus criteria was recorded for each ward round entry in yes/no format (Table 1).

The audit was repeated approximately two months following the introduction of the proforma between May 10, 2021, and 24, 2021. A two-month delay was chosen so the repeat audit accurately reflected proforma use in the medium to longer term, as this period spanned the rotation of junior medical staff.

Analysis of proforma use

**Figure 2:**
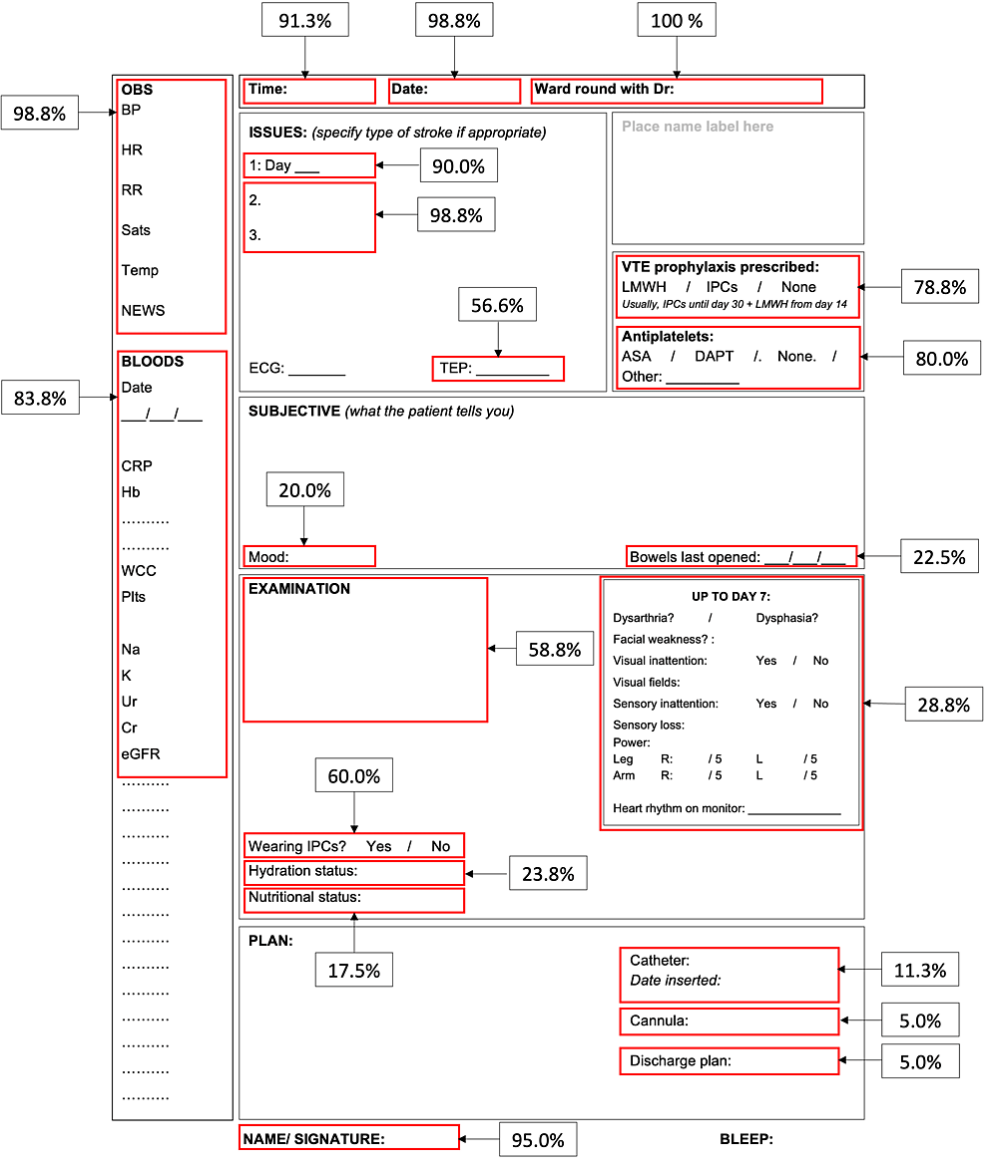
Annotated proforma showing the percentage of ward rounds documented using the proforma completing different sections. OBS: observations; BP: blood pressure; HR: heart rate; RR: respiratory rate; Sats: oxygen saturations; Temp: temperature; NEWS: national early warning score; CRP: c-reactive protein; Hb: hemoglobin; WCC: white cell count; Plts: platelets; Na: sodium; K: potassium; Ur: urea; Cr: creatinine; eGFR: estimated glomerular filtration rate; LMWH: low molecular weight heparin; VTE: venous thromboembolism; IPCs: intermittent pneumatic compression devices; ASA: aspirin; DAPT: dual antiplatelet therapy; ECG: electrocardiogram; TEP: treatment escalation plan; R: right; L: left

To further characterize patterns of use, the proforma was divided into sections (Figure 2). This included sections associated with consensus criteria parameters, for example, time, date, and observations, and sections not corresponding directly to the consensus criteria, including mood and documentation of the current TEP. The use of each section was recorded in yes/no format for all ward rounds using the proforma for documentation following its introduction.

MDT feedback

Feedback was obtained from the MDT regarding the usefulness and clarity of the proforma via an anonymous online questionnaire during the repeat audit period (between May 10, 2021, and 24, 2021). The questionnaire comprised a combination of Likert scale, multiple choice, and free-text questions.

## Results

Documentation standards pre-proforma

**Figure 3:**
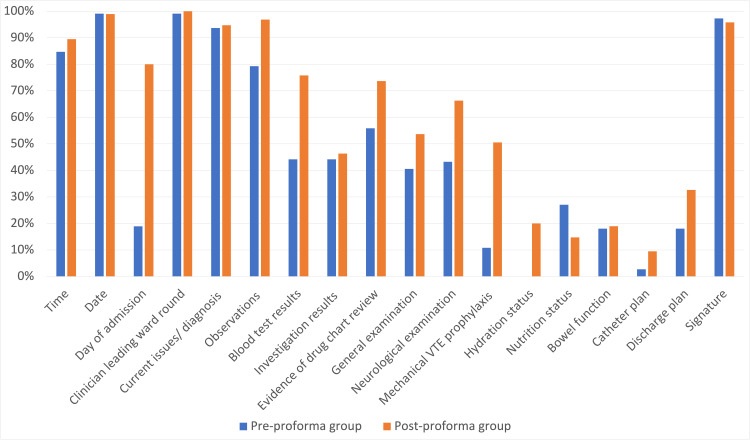
Percentage of ward round entries documenting consensus criteria before and after introduction of the proforma. VTE: venous thromboembolism

A total of 111 ward round entries were reviewed during the initial audit (between February 1, 2021, and February 15, 2021), before the introduction of the proforma. Ninety-five ward round entries were reviewed following the introduction of the proforma (between May 10, 2021, and 24, 2021). Prior to the introduction of the proforma, documentation of the date (99.1%), clinician leading the ward round (99.1%), signature of the documenting clinician (97.3%), and clinical issues/diagnosis (93.7%) were high. The least frequently documented parameters were discharge plan (18.0%), bowel function (18.0%), presence/absence of mechanical venous thromboembolism (VTE) prophylaxis (10.8%), and catheter plan (2.7%). No entries included documentation of hydration status (Figure 3).

Improvements in documentation following proforma introduction

A total of 84.2% (80/95) of ward round entries were documented using the proforma following its introduction and mean documentation of all criteria improved from 48.7% to 62.1%. All parameters improved except for nutritional status and signature, which fell from 27.0% to 14.7% and 97.3% to 95.8%, respectively (Figure 2). The greatest improvements were seen in the documentation on the day of admission (increased from 18.9% to 80.0%), blood test results (44.1-75.8%), neurological examination (43.2-66.3%), and presence/absence of mechanical VTE prophylaxis (10.8-50.5%).

Analysis of proforma use

Of ward rounds using the proforma for documentation, the most frequently completed sections were the clinician leading the ward round (100%), date (98.8%), clinical issues/diagnosis (98.8%), and observations (98.8%) (Figure 2). The least completed sections were catheter plan (11.3%), cannula plan (5.0%), and discharge plan (5.0%). The percentage of ward rounds using the proforma for documentation that completed the neurological examination section (28.8%) and discharge plan section (5.0%) was less than the overall documentation rate of these parameters anywhere on the ward round entry, 66.3% and 32.6%, respectively.

MDT feedback

At least 39 MDT members were invited to complete the anonymous online survey. A total of 11, 28.2% of those invited, responded, including senior medical staff (2), junior medical staff (7), and nursing staff (2). All respondents were aware of the new ward round proforma and the majority felt it was either very easy (54.5%) or easy (27.3%) to find in the patient notes. All respondents reported that the proforma made documentation of patients’ medical issues more clear (81.8%) or much more clear (18.2%), and the majority agreed that key patient information including blood tests, scan results, and examination findings was easier (45.5%) or much easier (45.5%) to find in the patient notes, and that documentation of patients’ medical plans was more clear (27.3%) or much more clear (36.4%). Junior medical staff felt the proforma was very easy (44.4%) or easy to use (55.6%). The majority also felt the proforma had made ward-round documentation faster (44.4%) or much faster (11.1%). Free-text feedback about the proforma was positive and praised its clear structure, stating that it served as a prompt to check key information including VTE prophylaxis prescription, electrocardiograms (ECGs), and blood results. Respondents also provided suggestions to improve the proforma layout, including increasing the size of the neurological examination box and adjusting the placement of the column containing blood results.

## Discussion

Introduction of the proforma resulted in improvement in documentation of the consensus criteria, with the greatest improvement seen in documentation of blood test results, day of admission, drug chart review (including antiplatelet prescription and pharmacological VTE prophylaxis), and presence/absence of mechanical VTE prophylaxis. More modest improvement was seen in documentation of observations, neurological examination, general examination, hydration status, and discharge plan, however, the neurological examination and discharge plan sections of the proforma were infrequently used. Feedback from the MDT was generally positive, with respondents reporting that the proforma made ward-round documentation clearer and easier to find in patient notes.

The consensus documentation criteria generated in this QI project are broadly similar to those employed in similar studies, including observations, examination findings, and diagnosis [4,6,7]. Some criteria are more specific to stroke patients, including documentation of mechanical VTE prophylaxis use, hydration status, and nutrition status. Dehydration is an important consideration following stroke; two-thirds of stroke patients become dehydrated during admission [15]. Dehydration is associated with poorer outcomes following stroke [16] and adequate fluid and nutrition reduce mortality [17].

The proforma design resembles the traditional SOAP (subjective, objective, assessment, plan) framework utilized in similar QI projects [2,18]. This is an evidence-based structured approach recommended by the RCP [1] that facilitates problem-orientated medical record keeping [2]. Some features of the proforma design are more specific to stroke patients including space to document antiplatelet prescription and a box for neurological examination modeled on the National Institute of Health Stroke Scale examination [19]. The proforma also included sections to record mood and note the treatment escalation plan (TEP); although these parameters were not recorded in the initial audit, 20.0% and 56.5%, respectively, of ward rounds documented using the proforma completed these sections.

The most important implication of these results is more comprehensive patient assessment during ward rounds. This is suggested by the considerable improvements seen in documentation of neurological examination and presence/absence of mechanical VTE prophylaxis, and more modest improvements in documentation of drug chart review, general examination, and hydration status. Daily physician ward rounds are associated with reduced mortality following stroke [17], and better ward round documentation has been associated with better patient outcomes [1,4], so these findings suggest a positive impact of the proforma on patient care.

MDT feedback was positive, suggesting the ward round proforma has made the documentation of medical issues and management plans clearer. Previous QI studies have suggested positive perceptions of ward round checklists amongst junior medical staff [4]. A smaller number of studies have collected broader MDT feedback regarding structured ward round checklists, which has been similarly positive [14,20].

Several factors may explain the low rates of documentation of discharge plan, nutritional status, hydration status, bowel function, and catheter plan. Discussions around discharge planning may not have been appropriate for every patient, depending on numerous factors including stroke acuity, other active medical issues, and social circumstances. Hydration and nutritional status may have been poorly documented due to uncertainty among junior medical staff about the level of detail required. Data about the number of patients who were catheterized during admission were not collected. However, patients were not routinely catheterized on admission to the stroke wards, therefore the low completion rate of the catheter section of the proforma likely reflects a low percentage of patients with a urinary catheter during their admission. Furthermore, information about catheter care and bowel function was primarily nurse-held, and nursing documentation about these parameters may not have been routinely reviewed on the medical round.

Using the results of this study, including survey feedback provided by the MDT, an updated proforma was designed with changes including multiple choice options for nutrition and hydration status, and removal of the catheter and discharge planning sections (Appendix). The size of the neurological examination box was increased as, although 71.3% of ward rounds documented a neurological examination following introduction of the proforma, only 28.8% used the section provided, with survey feedback suggesting this was due to insufficient space. The project was handed on to subsequent rotations of junior medical staff for repeat audit.

This study is limited as it does not report the direct impact of improved documentation on patient outcomes. Furthermore, evaluating documentation fairly between the initial and repeat audit following proforma introduction was challenging for some parameters. For example, any evidence of drug chart review was accepted as sufficient for the initial audit, whereas the majority of ward rounds reviewed during the repeat audit documented VTE prophylaxis prescription (78.8%) and antiplatelet prescription (80.0%) in the relevant proforma section. These results, therefore, are likely to under-represent the impact of the proforma on the quality and specificity of ward-round documentation.

## Conclusions

In conclusion, we report the impact of introducing a ward round proforma in a specialist stroke unit in the United Kingdom for the first time. This resulted in improvements in the quality and consistency of documentation which suggested more comprehensive patient assessment during ward rounds. The greatest improvements were seen in documentation of blood test results, neurological examination, presence of mechanical prophylaxis, and day of admission. Documentation of nutrition status fell and documentation of bowel function, hydration status, and catheter plan remained disappointingly low, although improved. We have introduced an amended proforma using the data presented and MDT feedback.

## Figures and Tables

**Figure 4:**
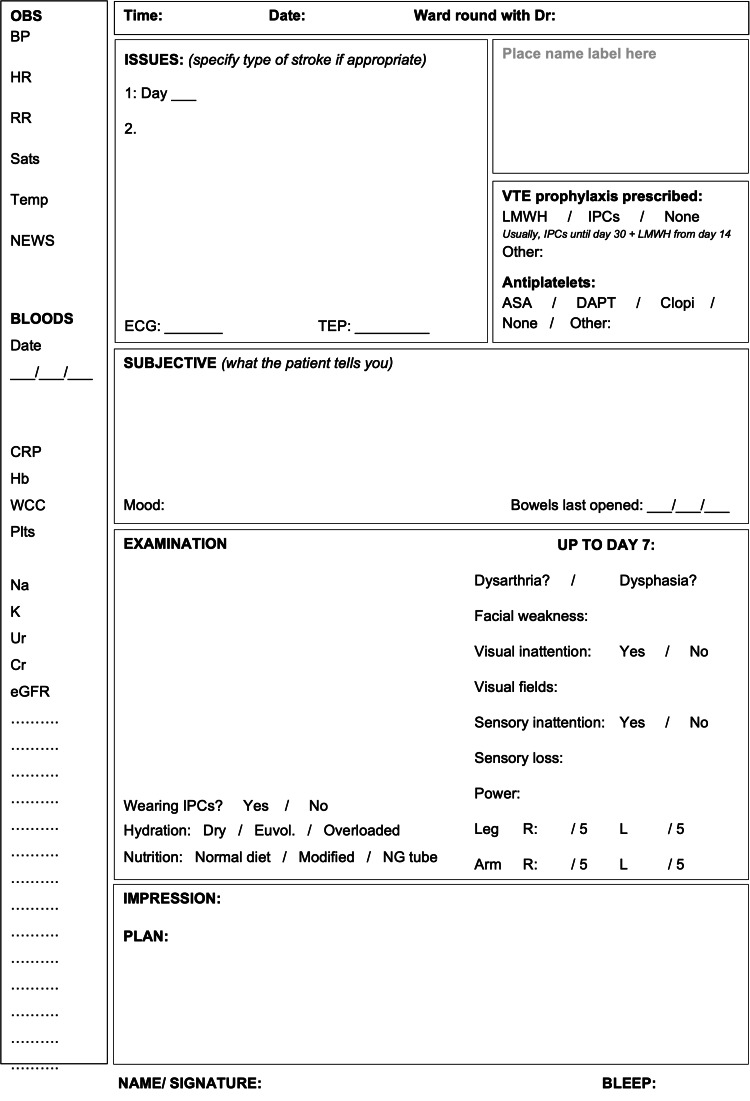
Revised proforma design. OBS: observations; BP: blood pressure; HR: heart rate; RR: respiratory rate; Sats: oxygen saturations; Temp: temperature; NEWS: national early warning score; CRP: c-reactive protein; Hb: hemoglobin; WCC: white cell count; Plts: platelets; Na: sodium; K: potassium; Ur: urea; Cr: creatinine; eGFR: estimated glomerular filtration rate; LMWH: low molecular weight heparin; VTE: venous thromboembolism; IPCs: intermittent pneumatic compression device; ASA: aspirin; DAPT: dual antiplatelet therapy; Clop: clopidogrel; ECG: electrocardiogram; TEP: treatment escalation plan; R: right; L: left; Euvol: euvolemic; NG: nasogastric
